# Genome-wide transcriptome analysis reveals the diversity and function of long non-coding RNAs in dinoflagellates

**DOI:** 10.1093/nargab/lqae016

**Published:** 2024-02-10

**Authors:** Yibi Chen, Katherine E Dougan, Quan Nguyen, Debashish Bhattacharya, Cheong Xin Chan

**Affiliations:** The University of Queensland, School of Chemistry and Molecular Biosciences, Australian Centre for Ecogenomics, Brisbane, QLD 4072, Australia; The University of Queensland, School of Chemistry and Molecular Biosciences, Australian Centre for Ecogenomics, Brisbane, QLD 4072, Australia; The University of Queensland, Institute for Molecular Bioscience, Brisbane, QLD 4072, Australia; Rutgers University, Department of Biochemistry and Microbiology, New Brunswick, NJ 08901, USA; The University of Queensland, School of Chemistry and Molecular Biosciences, Australian Centre for Ecogenomics, Brisbane, QLD 4072, Australia

## Abstract

Dinoflagellates are a diverse group of phytoplankton, ranging from harmful bloom-forming microalgae to photosymbionts of coral reefs. Genome-scale data from dinoflagellates reveal atypical genomic features, extensive genomic divergence, and lineage-specific innovation of gene functions. Long non-coding RNAs (lncRNAs), known to regulate gene expression in eukaryotes, are largely unexplored in dinoflagellates. Here, using high-quality genome and transcriptome data, we identified 48039 polyadenylated lncRNAs in three dinoflagellate species: the coral symbionts *Cladocopium proliferum* and *Durusdinium trenchii*, and the bloom-forming species, *Prorocentrum cordatum*. These lncRNAs have fewer introns and lower G+C content than protein-coding sequences; 37 768 (78.6%) are unique with respect to sequence similarity. We classified all lncRNAs based on conserved motifs (*k*-mers) into distinct clusters, following properties of protein-binding and/or subcellular localisation. Interestingly, 3708 (7.7%) lncRNAs are differentially expressed under heat stress, algal lifestyle, and/or growth phase, and share co-expression patterns with protein-coding genes. Based on inferred triplex interactions between lncRNA and putative promoter regions, we identified 19 460 putative gene targets for 3721 lncRNAs; 907 genes exhibit differential expression under heat stress. These results reveal, for the first time, the diversity of lncRNAs in dinoflagellates and how lncRNAs may regulate gene expression as a heat-stress response in these ecologically important microbes.

## Introduction

Dinoflagellates are a diverse group of microbial eukaryotes that are ubiquitous in marine and freshwater environments. These taxa range from parasites, symbionts of various organisms in coral reefs, to free-living species, of which some form harmful algal blooms that have serious implications for human health. Dinoflagellates in the family Symbiodiniaceae are known for their role as symbionts that supply essential nutrients and fixed carbon to coral hosts and other coral reef organisms (1). A periodic increase in ocean surface temperature can lead to carbon-flow disruption, photoinhibition, and breakdown of the coral-dinoflagellate symbiosis (i.e. coral bleaching) (2), putting corals at risk of starvation, disease, and eventual death. Another key ecological role of dinoflagellates is as bloom-forming species that cause ‘red tides’, producing toxins often lethal to marine life in large doses and/or accumulated by shellfish. When these toxins are ingested by human when consuming contaminated seafood, they may lead to paralytic or diarrhetic poisoning (3). Harmful blooms of dinoflagellates have been associated with warm waters, and some species are known to have expanded their habitats to the temperate oceans (4). For these reasons, and in light of warming oceans due in part to global climate change, the molecular response of dinoflagellates to heat stress has been a key focus of omics research.

Dinoflagellates have large, complex genomes up to ∼200 Gb in size, with features atypical of eukaryotes such as DNA packaged in permanently condensed chromosomes (5–7). The available genome data from dinoflagellates have revealed extensive genomic divergence, and the contribution of lifestyle to functional and structural diversification of genes and genomic elements (8–10), including distinct phylogenetic signals when comparing coding and non-coding sequences (11).

Earlier studies of transcriptome data identified: (a) differentially expressed genes in dinoflagellates relative to lifestyle (12), and under biotic and abiotic stresses (13–16), (b) the *trans*-splicing of spliced leader sequences at the 5′-end of mature mRNAs (17), (c) high diversity of transcript isoforms (18), and, more recently in combination with genome data, (d) the abundance of protein-coding sequences (8,19–25) and (e) the differential editing of mRNAs (10,26) and differential exon usage (27) under distinct growth conditions. In most cases, these studies used standard RNA-Seq approaches for eukaryotes to generate transcriptome data, whereby mRNAs with polyadenylated tails are retained, and the encoded protein-coding genes are analysed. Many non-coding RNAs (ncRNAs) with polyadenylated tails would exist in these datasets (28), but have been largely ignored.

Our current understanding of how gene expression is regulated in dinoflagellates remains limited. In eukaryotes, transcription factors (TFs) bind to specific DNA regions in the genome, thereby enhancing or suppressing transcription of the associated gene(s). TFs make up ∼4% of the proteome in yeast, and ∼8% of the proteome in plants and mammals (29). In comparison, proteins that contain DNA-binding domains account for only 0.15%–0.30% of all proteins in dinoflagellates (30,31). In an independent analysis of the dinoflagellates *Lingulodinium polyedra* and *Fugacium kawagutii*, ∼60% of putative TFs (i.e. DNA-binding proteins) contain a cold shock domain, and they tend to bind to RNA instead of specific double-stranded DNA as expected for canonical TFs (32). This observation hints at the role of RNAs in regulating gene expression in dinoflagellates.

The regulatory role of ncRNAs in gene expression has been demonstrated in diverse eukaryotes (33,34). The two most common types of ncRNAs with regulatory functions are microRNAs (21–23 nt in length) and long non-coding RNAs (lncRNAs; >200 nt (35,36), or more recently, recognised sequences of length >500 nt (34)). Target mRNA molecules are bound by microRNAs, which suppress translation of the transcript into protein and/or cause their degradation. In dinoflagellates, microRNAs have been associated with the regulation of protein modification, signalling, gene expression, sulfatide catabolism (37), and amino acid metabolism (38), as well as the molecular responses to DNA damage (39) and symbiosis (21). In comparison, lncRNAs in eukaryotes regulate gene expression by altering chromatin structures, transcription of genes, molecular scaffolding, and assembly of RNA-protein compartments (40). For instance, they can serve as molecular scaffolds that link TFs to target genes, thereby altering their transcription (41). The genomic loci for lncRNA binding have also been associated with the recruitment of chromatin-modifying complexes, thus changing the accessibility of nearby genes or the entire chromosome (42). To date, lncRNAs in dinoflagellates remain uncharacterised and largely unexplored.

Here, we investigate the conservation and functional role of lncRNAs in dinoflagellates relative to distinct growth conditions using high-quality genome assemblies and transcriptome datasets from three taxa: *Cladocopium proliferum* SCF055 (25,43) (an isolate formerly identified as *Cladocopium goreaui* SCF055 (44)), *Durusdinium trenchii* CCMP2556 (12,27) and *Prorocentrum cordatum* CCMP1329 (10), all generated under heat-stress experiments (Table 1). *C. proliferum* (44) and the naturally thermotolerant *D. trenchii* (1,45,46) are two key symbiont species for reef-building corals, particularly in the Indo-Pacific region, whereas *P. cordatum* is a globally distributed, invasive bloom-forming species (47,48) known to tolerate a broad range of salinities and temperatures, and exhibiting an increased bloom frequency. Due to the different designs of the experiments associated with these independently generated datasets, these data allow for assessment of heat-stress response at slightly different resolutions: (a) early- versus late-stage response (*C. proliferum*), (b) free-living versus symbiotic stages (*D. trenchii*) and (c) exponential versus stationary growth phases (*P. cordatum*).

**Table 1.** The three core dinoflagellate transcriptome datasets used in this study

			Transcriptome dataset					
Isolate	Estimated genome size	Genome assembly	Condition factors	Number of samples	Number of bases (Gbp)	Number of uniquely mapped reads	% assembled genome transcribed	Source
*Cladocopium proliferum* SCF055 (formerly *Cladocopium goreaui* SCF055)	1.30 Gb	(25)	Treatment T1 (early-stage response to 32°C)	4	10.79	80 286 755	7.08	(43)
			Treatment TE (late-stage response to 32°C)	4	11.35	86 223 788	7.61	
			Control: 26°C	8	22.25	167 140 391	7.19	
*Durusdinium trenchii* CCMP2556	1.05 Gbp	(27)	Treatment 34°C (free-living)	4	42.97	79 752 559	11.09	(12)
			Treatment 34°C (symbiotic)	4	52.97	104 474 522	10.27	
			Control: 28°C (free-living)	4	50.50	104 626 936	11.03	
			Control: 28°C (symbiotic)	4	47.39	95 486 315	9.32	
*Prorocentrum cordatum* CCMP1329	4.75 Gbp	(10)	Mild heat stress at 26°C (exponential)	3	97.00	487 707 438	3.97	(10)
			Mild heat stress at 26°C (stationary)	3	99.99	358 069 375	4.31	
			Severe heat stress at 30°C (exponential)	3	102.81	471 062 368	4.36	
			Severe heat stress at 30°C (stationary)	3	99.50	470 249 140	4.10	
			Control: 20°C (exponential)	3	91.48	431 667 151	4.02	
			Control: 20°C (stationary)	3	102.60	453 517 396	3.58	

## Materials and methods

### Data

We used published genome assemblies of *Cladocopium proliferum* SCF055 (25), *Durusdinium trenchii* CCMP2556 (27) and *Prorocentrum cordatum* CCMP1329 (10), for which the associated transcriptome data are also available. For each of these taxa, a core transcriptome dataset, designed for heat stress experiments (Table 1) was selected to minimise technical biases. For the *C. proliferum* dataset that consists of 24 samples (43), 16 representing T1 (early response) and TE (late response) were included (Table 1 and Supplementary Table S1); the remaining eight were generated under control conditions that did not involve heat stress.

### Identification of lncRNAs

We developed a customised workflow to identify putative lncRNAs (https://github.com/YibiChen/LncRNAPredictor and https://doi.org/10.5281/zenodo.10558662) using genome and transcriptome data of dinoflagellates (Supplementary Figure S1). For each taxon, all raw RNA-Seq reads from the core transcriptome dataset were processed and adapter-trimmed using Fastp v0.23.2 (49), and assembled into transcripts *de novo* using Trinity v2.9.1 (50). For *C. proliferum*, due to the low data yield of the core dataset (Table 1), raw reads from other available transcriptome data (51,52) (Supplementary Table S1) were also included in this step to maximise recovery. The resulting transcripts were then mapped to their corresponding genome sequences with Minimap v2.24 (53) for which the code was modified to recognise alternative splice sites of dinoflagellate genes, and further assembled using PASA v2.3.3 (54). Because the RNA-Seq data are generated from polyadenylated transcripts, these PASA-assembled transcripts represent polyadenylated protein-coding genes and lncRNAs. Protein-coding genes, i.e. transcripts that overlap (minimum 1 bp) with one or more exonic regions, were identified and removed. The coding potential of the remaining transcripts were assessed using three methods: CPC2 (55) at default setting, and FEELnc (56) independently at *-m intergenic* and *-m shuffle* modes. Transcripts classified as non-coding by all three methods were retained. We then removed transcripts that share significant sequence similarity to known Pfam protein domains (BLASTx, *p ≤*10^−5^); the remainder represent potential lncRNA candidates. To reduce redundancy, overlapping sequences were clustered based on genome location, among which the longest sequence was selected as the representative lncRNA from each cluster. To identify lncRNAs at high confidence for each isolate, we mapped the RNA-Seq reads from the core transcriptome dataset to the corresponding genome sequences using HISAT v2.2.1 (57), and counted the number of uniquely mapped reads using *featureCounts* (58). Only lncRNA candidates (identified above) supported by 10 or more samples (each with 10 or more uniquely mapped reads), were considered as high-confidence, putative lncRNAs and used for downstream analyses (Supplementary Figure S1). A total of 48 039 putative lncRNAs were identified in this way from all three taxa (Table 2). For clarity of presentation, hereinafter we define *gene* strictly as a protein-coding gene, excluding lncRNAs.

**Table 2.** The lncRNAs identified in the three dinoflagellate taxa, their statistics relative to genes and the associated lncRNA:gene ratios

Taxon	*Cladocopium proliferum* SCF055			*Durusdinium trenchii* CCMP2556			*Prorocentrum cordatum* CCMP1329		
Metric	lncRNA	Gene	Ratio	lncRNA	Gene	Ratio	lncRNA	Gene	Ratio
Counts	13 435	45 322	0.30	7036	55 799	0.13	27 568	85 849	0.32
Mean length (bp)	450	2018	0.22	447	1647	0.27	493	2798	0.18
% G+C	48.8	54.2	0.90	51.7	55.7	0.93	61.7	65.9	0.94
Mean number of introns per lncRNA/gene	0.8	16.3	0.05	2.0	15.7	0.13	0.39	9.8	0.04
% lncRNAs/genes that contain introns	23.0	95.9	0.24	51.7	93.1	0.56	27.3	83.7	0.33

### Identification of homologous lncRNA based on overall sequence similarity

Among the 48 039 lncRNAs identified above, we investigated homologous sets based on sequence similarity using OrthoFinder v2.3.8 (59). To identify the putative functions of lncRNAs, we used BLASTn v2.11.0+ (60) to search the 48 039 lncRNAs as queries against the high-quality curated database of human lncRNAs, FANTOM CAT (89 998 sequences tagged as *lv3_robust*) (61), all lncRNA sequences in the NCBI nr database (17 107; acquired on 15 August 2022) and the NONCODE v6.0 database (62). This alignment-based approach enables inference of homology based on overall shared similarity of the lncRNA sequences.

### Identification of homologous lncRNA based on conserved motifs

In an alternative approach, we assessed homologous lncRNAs using *k*-mers. Here, a *k*-mer is a short, sub-sequence of pre-defined length *k*, derived from the putative lncRNA sequences. All possible *k*-mers derived from a lncRNA sequence represent its *k*-mer profile. The extent of shared *k*-mers between two lncRNA sequences (i.e. the similarity of the *k*-mer profiles) reflect their extent of sequence similarity (and thus potential homology). We used seekr (63) to group the 48 039 putative lncRNAs into 10 clusters based on the *k*-mer profiles of lncRNAs, at *k* = 4. Specifically, we used *seekr_k-mer_count* to count the number of distinct 4-mers in each lncRNA and normalised the count using *seekr_norm_vector* against 4-mers from all lncRNAs as background. The normalised 4-mer profiles were then used to calculate an adjacency matrix using *seekr_pearson* for each possible pair of lncRNAs. Based on hierarchical clustering, we used fcluster (*t = 10, criterion=‘maxclust’*) available from the SciPy Python library (64) to group the lncRNAs into 10 clusters; we refer these to as the *k*-mer-based lncRNA clusters. This method is more scalable to the large datasets we used in this study compared to the Louvain algorithm used in Kirk *et al.* (63), and was found to yield similar results in the same study.

### Analysis of differential expression of lncRNAs

For each core transcriptome dataset, filtered read count for transcripts (including both lncRNAs and genes) were normalised using the getVarianceStabilizedData implemented in DESeq2 (65). To assess variation of samples within the dataset, the normalised counts were used to derive pairwise Euclidean distance between samples, from which the samples were grouped using hierarchical clustering. Samples that did not group with other replicates were considered outliers and excluded from downstream analysis. We used DESeq2 (65) to identify differentially expressed transcripts (i.e. lncRNAs and genes) related to heat stress, lifestyle, and/or growth phase. To minimise false positives, we adopted a stringent threshold for inferring differential expression, whereby we consider transcripts with an adjusted (66) *p* ≤ 0.01 and absolute (log_2_[fold-change]) ≥2 to be differentially expressed.

### Co-expression analysis

We performed weighted gene co-expression network analysis (WGCNA) (67) to assess transcript co-expression. The soft-thresholding power parameter (*T*), which assigns weighting of transcript co-expression, was carefully selected for each core transcriptome dataset. This was guided by the scale-free topology fit index (*I*), calculated from the normalised read counts of transcripts using the pickSoftThreshold function. We used *T* = 18 in all three datasets for different reasons. For *C. proliferum* and *P. cordatum*, *T* = 18 is the smallest value that gave rise to *I* > 0.8 (representing a good fit of the network). For *D. trenchii*, *I* was < 0.8 for *T* between 10 and 30; we therefore used *T* = 18 as recommended for signed networks with fewer than 20 samples (https://horvath.genetics.ucla.edu/html/CoexpressionNetwork/Rpackages/WGCNA/faq.html).

Next, we calculated the topological overlap matrix for signed network using bicorr correlation (*maxPOutliers = 0.1, pearsonFallback=‘individual’*) to assess co-expression similarity and adjacency, from which the dissimilarity was used to group the transcripts using hierarchical clustering (*method = complete*). Highly co-expressed transcripts were identified by ‘cutting’ the branches using dynamicTreeCut (*deepSplit = 2, pamRespectsDendro = FALSE, minModuleSize = 30*). We then clustered module eigengenes (*method = average*) and merged those with high expression correlation (eigengene correlation ≥ 0.9).

To assess the correlation between *k*-mer-based lncRNA clusters independently for each WGCNA module, we tested whether lncRNAs from any of the 10 *k*-mer-based clusters were significantly over- or under-represented using Fisher's exact test (false discovery rate ≤ 0.01; two-tailed). To assess conserved motifs among a set of lncRNAs, we used MEME v5.5.3 (*-dna -nmotifs 3*) (68).

### Identification of lncRNA targets

We used Triplexator (69) to search for possible formation of triple-helical structures between a lncRNA and the putative promoter region of a gene following the Hoogsteen base pairing rules. We defined putative promoter regions as 1 kb upstream of a coding sequence following Lin et al. (21). Triplexator was run requiring a minimum length of 20 bp for the binding region and no more than 5% mismatches, with *-l 20 -e 5 -fr on -mrl 7 -mrp 3 -dc 5 -of 1*.

## Results and discussion

### LncRNAs have fewer introns and lower G+C-content than protein-coding genes

We adopted a stringent approach to identify putative lncRNAs, focusing on expressed RNAs transcribed from genome regions that do not overlap with exons. Briefly, we consider a transcript to be lncRNA if it: (a) mapped to the reference genome (at ≥95% sequence identity covering ≥75% of query) and (b) did not encode any Pfam protein domains, was (c) classified as non-coding by three methods for calculating protein-coding potential and (d) was expressed in 10 or more samples; see Materials and methods and Supplementary Figure S1.

Using this approach, we identified distinct lncRNAs in *C. proliferum* (13 435), *D. trenchii* (7036) and *P. cordatum* (27 568), with a combined total of 48 039 (Table 2). The number of lncRNAs in *P. cordatum* is more than twice of that in *C. proliferum* (genome size ∼1.5 Gb, 45 322 genes) (25), likely due to the larger genome size (∼4.75 Gb) and number of genes (85 849) in *P. cordatum* (10). However, proportionately, the number of lncRNAs is approximately one-third of that of genes in these two genomes, i.e. 0.30 in *C. goreaui* and 0.32 in *P. cordatum* (Table 2). The equivalent ratio is much smaller (0.13) in *D. trenchii*, which may be affected by whole-genome duplication implicated in this lineage (27).

**Figure 1.**
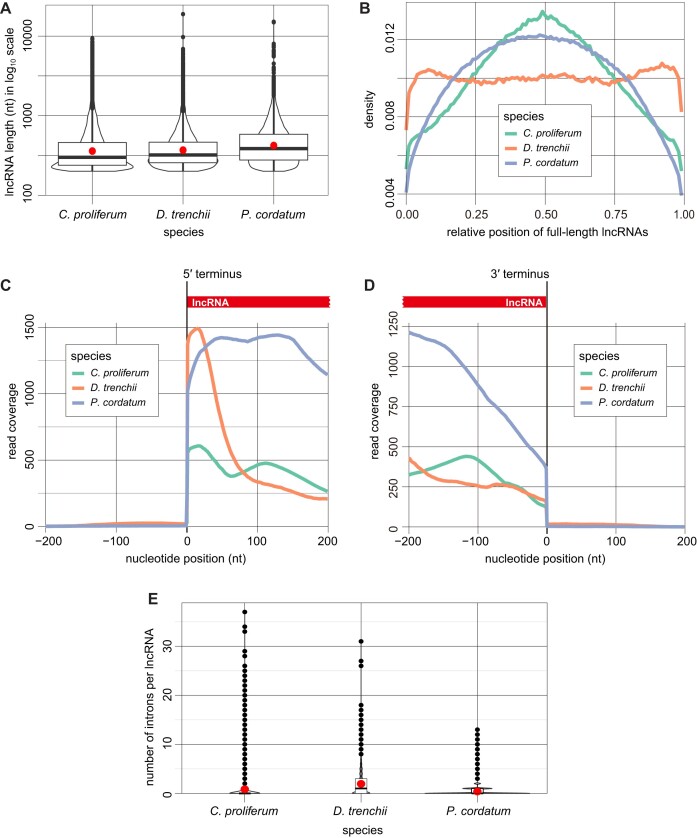
Dinoflagellate lncRNAs in *C. proliferum*, *D. trenchii* and *P. cordatum*. (**A**) The distribution of lncRNA lengths on a log_10_ scale with mean lengths indicated as red dots. (**B**) Density plot of RNA-Seq read coverage across the relative positions of full-length lncRNAs. (**C**) RNA-Seq read coverage of the ± 200 nt at the 5′-termini of lncRNAs. (**D**) RNA-Seq read coverage of the ± 200 nt at the 3′-termini of lncRNAs. (**E**) The number of introns per lncRNA with mean values indicated as red dots.

Among the three genomes, the mean length of lncRNAs (447–493 bp; Table 2 and Figure 1A) was shorter than that of genes (1657–2798 bp; Table 2). These lncRNA sequences are shorter than in other taxa (∼1 to >100 kb) (34), likely due to the RNA-Seq (short-read) transcriptome datasets used in this study, in which full-length lncRNAs may not have been recovered as readily as when using long-read technology (70). In addition, non-polyadenylated lncRNAs (34) would have been excluded due the standard poly-A enrichment step during the preparation of RNA-Seq sequencing libraries. Read coverage across the lncRNAs in full length (Figure 1B) revealed lower coverage in the 5′- and 3′ termini when compared to the middle region, indicating some evidence of RNA degradation near the termini. The near-uniform read coverage for the *D. trenchii* lncRNAs (Figure 1B) suggests a smaller extent of RNA degradation when compared to datasets from the other two taxa. If the impact of RNA degradation was severe in biasing our results, we expect lncRNAs in *D. trenchii* to be substantially longer than those from the other two taxa; this is not the case (Figure 1A). Furthermore, we observed drastic changes in read coverage at the 5′- (Figure 1C) and 3′-termini (Figure 1D) of all lncRNAs. These results suggest that most lncRNAs we identified in this study are likely to be full-length sequences.

The lncRNAs exhibited lower (0.90–0.94-fold) G+C-content than genes (e.g. mean 48.8% compared to 54.2% for *C. proliferum*; Table 2), and had fewer introns, e.g. mean 0.39 introns per lncRNA (Table 2 and Figure 1E) compared to mean value of 9.8 introns per gene in *P. cordatum* (Table 2). These numbers are comparable to earlier studies of eukaryotes, e.g. lncRNAs among the Brassicaceae plants are shorter and with a lower G+C-content when compared to genes (70), and those in zebra finch have fewer introns than do the genes (71). Adopting the approach by Stephens et al. (19), we found evidence of spliced leader *trans*-splicing in 2 and 121 lncRNAs respectively for *C. proliferum* and *P. cordatum*, none in *D. trenchii*, potentially due to the smaller number of transcripts in this dataset. This result supports the notion that spliced leader *trans*-splicing is a molecular machinery common to both lncRNAs and transcripts of (protein-coding) genes in dinoflagellates, although not universal to all transcripts (17,19).

### Most lncRNAs were transcribed in the same orientation as protein-coding genes

**Figure 2.**
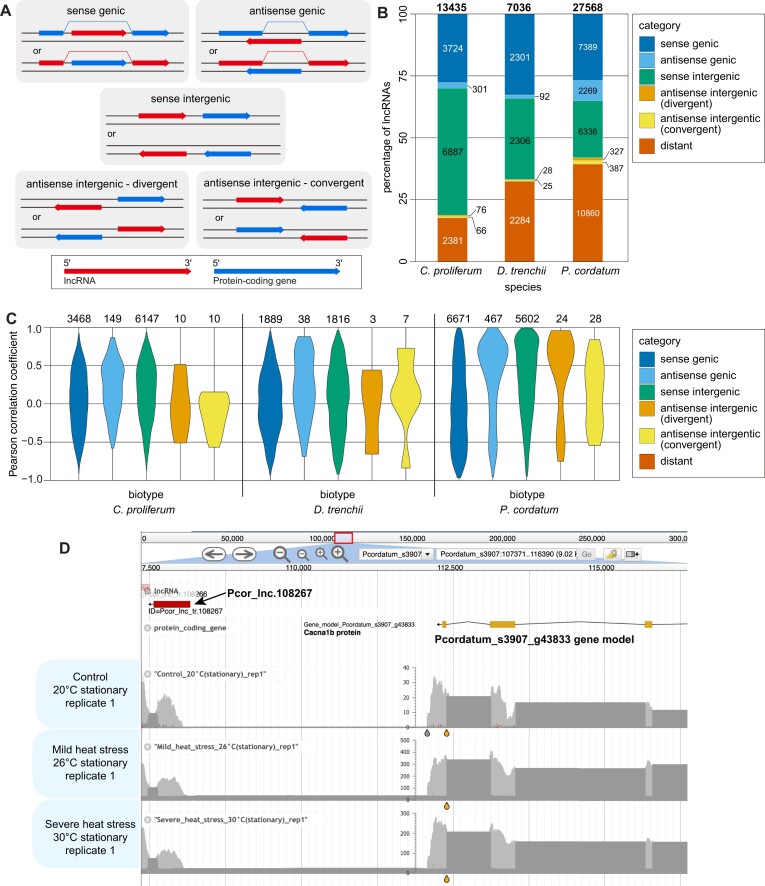
Location of lncRNAs relative to the nearest protein-coding genes in genomes. (**A**) Schematic diagram showing five distinct lncRNA biotypes based on their coding configuration relative to the nearest protein-coding gene on the double-stranded genome sequence. Shown for *C. proliferum*, *D. trenchii* and *P. cordatum*, (**B**) the percent and number of lncRNAs classified into each lncRNA biotype (including ‘distant’) as stacked bars with total number of lncRNAs shown on top and (**C**) Pearson correlation coefficients showing the correlation of expression between each pair of lncRNAs and the nearest corresponding gene, for each biotype. (**D**) Diagram showing the expression (based on uniquely mapped RNA-Seq reads) of a ‘sense intergenic’ *P. cordatum* lncRNA (Pcor_lnc.108267) and its corresponding gene (Pcordatum_s3907_g43833) under heat stress during stationary phase.

Many lncRNAs target genes located close to where the transcribed lncRNAs are found in the genome; these lncRNAs are referred to as *cis*-acting lncRNAs (72). In some species, these *cis*-acting lncRNAs and their target genes may display a consistent configuration of coding orientations. For instance, gene and antisense lncRNAs expression are globally anti-correlated in yeast (73). A similar anti-correlation is observed in the malaria parasite *Plasmodium falciparum* (74) which is in the phylum Alveolata that includes dinoflagellates. Therefore, the configuration of the lncRNAs relative to their corresponding genes that are closely located on a genome can provide evidence of their putative targets and potential regulatory mechanism for expression. To investigate this aspect, we followed Luo et al. (75) to consider a gene located closest to a lncRNA with 5 kb upstream or 5 kb downstream (whichever is closer) as the corresponding gene for the lncRNA. Based on the coding orientation of the lncRNA and the corresponding gene, we classified the lncRNAs broadly into five biotypes (Figure 2A): (a) ‘sense genic’, whereby the lncRNA is encoded in the introns of the gene or spanned the entire gene in the same orientation, (b) ‘antisense genic’, whereby lncRNAs that overlap the genomic span (i.e. exon or introns) of the gene on the opposite strand, (c) ‘sense intergenic’, whereby the intergenic lncRNA and the gene are encoded in the same orientation, (d) ‘antisense intergenic – divergent’, whereby the intergenic lncRNA and the gene form a head-to-head configuration, and (e) ‘antisense intergenic (convergent)’, whereby the intergenic lncRNA and the gene form a tail-to-tail configuration. All other lncRNAs for which no gene(s) were identified within 5 Kbp upstream, 5 kb downstream, or in region extending beyond the tail end of a genome scaffold, were classified as ‘distant’. Because lncRNAs identified in this study do not share any exonic regions (Supplementary Figure S1), ‘sense genic’ lncRNAs (Figure 2A) are in intronic regions of genes encoded in the same orientation.

Figure 2B shows the percentage and number of lncRNAs in the distinct biotypes for each of the three dinoflagellate genomes. Sense lncRNAs (both ‘sense genic’ and ‘sense intergenic’) are dominant in these genomes, accounting for 10 611 (79.0%), 4607 (65.5%) and 13 725 (49.8%) lncRNAs respectively in *C. proliferum*, *D. trenchii* and *P. cordatum*. These numbers are in stark contrast to those for antisense lncRNAs, i.e. ‘antisense genic’, ‘antisense intergenic – divergent’, and ‘antisense intergenic – convergent’. For instance, the combined ‘antisense intergenic’ biotypes account only for 142 (1.1%), 53 (0.8%) and 714 (2.6%) lncRNAs in *C. proliferum*, *D. trenchii* and *P. cordatum* (Figure 2B). These results are consistent with the observation of unidirectionally encoded genes in dinoflagellate genomes (19,20,25), which now extends to lncRNAs. Although it is unclear that ‘sense genic’ lncRNAs would act in *cis* regulation (76), ‘sense intergenic’ lncRNAs are known to suppress or maintain the expression of nearby genes (77), representing useful candidates for further functional validation. Compared to *C. proliferum* (2381, 17.7%), we observed a greater extent of ‘distant’ lncRNAs in *D. trenchii* (2284, 32.5%) and in *P. cordatum* (10 860, 39.4%) (Figure 2B). This observation likely reflects the fragmented state of the genome assemblies, whereby assembly of the *D. trenchii* genome was impacted by a recent whole-genome duplication (27). The *P. cordatum* assembly is more fragmented due in part to its large size of ∼4.75 Gb (10) (Table 1).

We assessed the correlation of expression between lncRNAs and corresponding genes in each biotype, focusing on pairs for which both the lncRNA and the gene were expressed (Figure 2C). A Pearson correlation coefficient approximating 1.0 provides strong evidence of co-expression, whereas a coefficient approximating –1.0 indicates a strong anti-correlation. In general, we find no evidence of strong anti-correlation as observed in yeast or *Plasmodium*. For *P. cordatum*, we observed a slight anti-correlation among the 6671 ‘sense genic’ lncRNAs and their corresponding genes, and slight positive correlation among lncRNAs classified as ‘antisense genic’, ‘sense intergenic’ and ‘antisense intergenic – divergent’ (Figure 2C). These results indicate the impact of lncRNA expression in dinoflagellates with the expression of adjacent or overlapping genes in the genome, although we did not observe a common trend or bias for specific configuration of coding orientations among the three datasets that were analysed. However, these lncRNAs represent useful candidates for future investigations of gene regulation in dinoflagellates.

As an example (Figure 2D), we observe approximately 6- to 10-fold greater expression of a ‘sense intergenic’ lncRNA in *P. cordatum* (Pcor_lnc.108267) under heat stress during the stationary growth phase at 26°C and at 30°C, compared to the control at 20°C. This observation is not due to technical bias because the data yield from each of these samples are similar (Table 1). Interestingly, we observe a strong positive correlation (Pearson correlation coefficient = 0.94) between the expression of Pcor_lnc.108267 and the expression of the corresponding gene (Pcordatum_s3907_g43833) that encodes the voltage-dependent N-type calcium channel protein subunit alpha-1B (CACNA1B) (Figure 2D). This result indicates a potential regulatory role of the *cis*-acting lncRNA on the expression of the gene encoding CACNA1B in *P. cordatum* under heat stress. The encoded calcium channel protein, responsible for calcium transport and uptake, is also known to be up-regulated under heat stress in the coral *Acropora millepora* (78) and under light stress in the giant clam, *Tridacna squamosa* (79).

### LncRNAs exhibit conserved motifs despite sharing low overall sequence similarity

**Figure 3.**
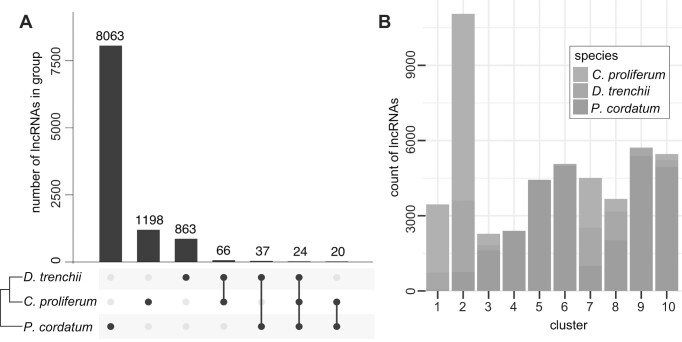
Homologous lncRNAs identified in the three dinoflagellate taxa based on (**A**) shared sequence similarity, and (**B**) conserved sequence motifs of *k*-mers.

To assess conservation of lncRNA sequences, we identified 2325 putative homologous sets from the 48 039 lncRNAs recovered in all three taxa based on sequence similarity. Most lncRNAs (37 768; 78.6%) were not assigned to any homologous sets (i.e. they are singletons). Among those assigned to a set, most (2291 of 2325 [98.5%] sets) represent highly duplicated lncRNAs within a genome, e.g. 1559 homologous sets implicating 8063 lncRNAs are specific to *P. cordatum* (Figure 3A). This result suggests that, in contrast to genes, lncRNA sequences are not highly conserved across taxa (80). However, few exceptions are known, such as the MALAT1 lncRNA with a highly conserved 3′-end processing module in animals and a functional role in cell cycle progression and cell migration in humans (81). Interestingly, two homologous sets implicating 24 lncRNAs were identified in all three taxa. Given the lack of lncRNA resources for non-model systems, we searched for these lncRNAs in the high-quality curated database of human lncRNAs, FANTOM CAT (61), and other lncRNAs from the NCBI nr database.

One of the two sets, containing 19 lncRNAs, shows significant sequence similarity (BLASTn, *E* ≤ 10^–5^) with the human lncRNA CATG00000009539.1 in FANTOM CAT, and the mouse (*Mus musculus*) lncRNA MSUR1 in the nr database; CATG00000009539.1 is an intergenic lncRNA expressed in small intestine cells (82), whereas MSUR1 is known to rescue cell death mediated by copper/zinc superoxide dismutase (83). When the search used the more-expansive lncRNA database of NONCODE version 6 (62), we found significant hits (BLASTn, *E* ≤ 10^−5^) from various species, including yeast, plants (*Arabidopsis thaliana* and maize), and other animals (*Caenorhabditis elegans*, zebrafish, and the fruit fly *Drosophila melanogaster*). Among the dinoflagellate sequences, the lncRNA of *P. cordatum* shared 79.78% identity with over 75.43% of the MSUR1 sequence in mouse. Although the function of these lncRNAs in dinoflagellates remains to be validated experimentally, these results indicate strong evidence for cross-phylum sequence conservation and putative homology with potential functions related to oxidative stress. In contrast, the other homologous set (comprising five lncRNAs recovered from in all three dinoflagellate genomes) does not share significant sequence similarity to any sequence in the databases we used.

Our results lend support to the lack of shared sequence similarity and/or contiguity among lncRNAs, as observed among homologous lncRNAs in other eukaryotes (84). For this reason, the alignment-free approach based on conserved *k*-mers (i.e. short motifs of length *k*) provides a good alternative for identifying lncRNAs with similar functions, specifically those containing motifs related to protein binding and subcellular localisation (63). Using this approach, we generated normalised *k*-mer profiles using all lncRNAs from the three genomes and calculated a pairwise adjacency matrix for all possible lncRNA pairs. Using hierarchical clustering, we classified all lncRNAs into 10 clusters based on their shared *k*-mer profiles (Figure 3B); those from *C. proliferum* and *D. trenchii* tend to group together (e.g. clusters 1 and 2), distinct from *P. cordatum* (e.g. clusters 4, 5 and 6). This observation suggests more conserved lncRNA motifs for putative protein binding and subcellular localisation between the coral symbionts *C. proliferum* and *D. trenchii* (both of family Symbiodiniaceae in order Suessiales), when compared to the free-living *P. cordatum* (order Prorocentrales), consistent with phylogenetic relationships among the three taxa.

### LncRNAs are differentially expressed in response to heat stress

**Figure 4.**
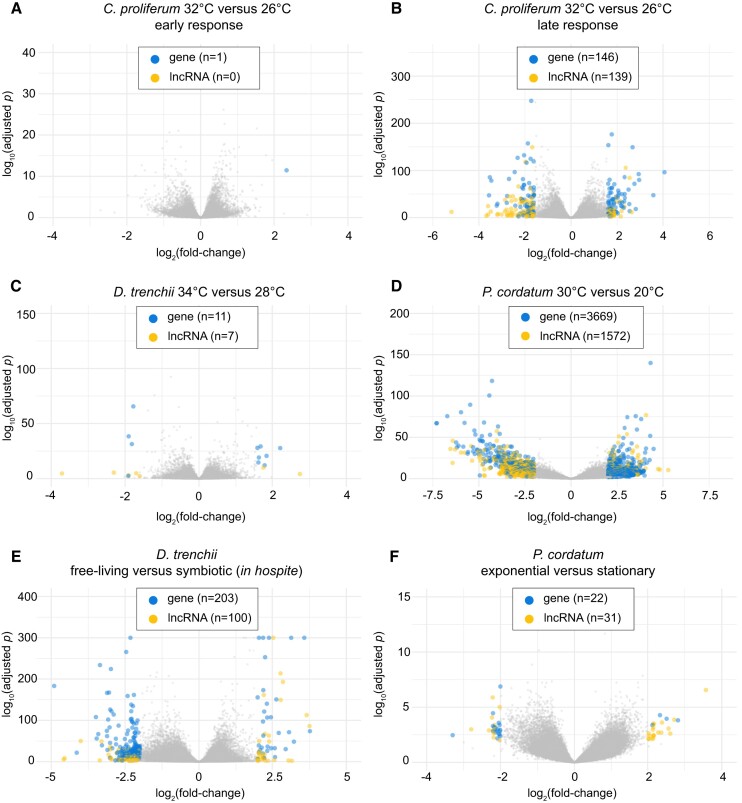
Differentially expressed genes and lncRNAs relative to heat stress, lifestyle, and growth phase. Volcano plots are shown for (**A**) *C. proliferum* (early-stage response T1: 32 versus 26°C), (**B**) *C. proliferum* (late-stage response TE: 32 versus 26°C), (**C**) *D. trenchii* (34 versus 28°C), (**D**) *P. cordatum* (30 versus 20°C), (**E**) *D. trenchii* (free-living versus symbiotic stage) and (**F**) *P. cordatum* (exponential versus stationary phase). For each panel, the *x*-axis represents fold-change of transcript expression (in log_2_ scale), and the *y*-axis represents the significance of difference in adjusted *P*-value (in log_10_ scale). For simplicity, log_10_(adjusted-*p*) >300 is shown as 300. Differentially expressed genes (blue) and differentially expressed lncRNAs (yellow) were noted, and their numbers are shown for each panel.

Using the transcriptome dataset for each taxon, we identified differentially expressed (DE) lncRNAs in response to heat stress, growth phase, and/or lifestyle (Figure 4), based on a stringent criterion (see Materials and Methods); in combination, we observed 3708 DE lncRNAs. In *C. proliferum*, no DE lncRNAs were observed during early-stage heat stress (T1) relative to controls (Figure 4A) compared to 139 DE lncRNAs at late-stage heat stress (TE; Figure 4B), a pattern which is consistent with the DE genes (1 in Figure 4A, 146 in Figure 4B). Similarly, in an experiment that measured the heat-stress response of *D. trenchii* at day 3 of temperature ramping to 34°C, few DE lncRNAs (7) and genes (11) were observed (combining free-living and symbiotic stages; Figure 4C). Interestingly, we identified three orders of magnitude greater DE lncRNAs (1572) and genes (3669) under heat stress in *P. cordatum* (combining both exponential and stationary growth phases; Figure 4D) than those observed in the two symbiotic species. Whether the difference we observed among the three taxa is related to their distinct ecological niches is unclear due to the different experimental designs used to generate the data, including sequencing depth. We also observed DE lncRNAs relative to free-living versus symbiotic stages in *D. trenchii* (Figure 4E), and to the distinct growth phases of *P. cordatum* (Figure 4F), as similarly observed for the DE genes in earlier studies (12,27). A greater transcriptional response to heat stress was observed in *P. cordatum* during stationary growth (longer exposure to heat stress) than in the exponential phase (shorter exposure to heat stress). Our results, also independently observed at distinct growth phases of *P. cordatum* (Supplementary Figure S2) and in combination with the previous observations (10,43), suggest a greater transcriptional response in dinoflagellates under prolonged heat stress. This may explain in part why in some earlier studies (85,86) that assessed transcriptional changes within a shorter treatment period found few DE transcripts in dinoflagellates.

### LncRNAs of similar *k*-mer profiles tend to be co-expressed

**Figure 5.**
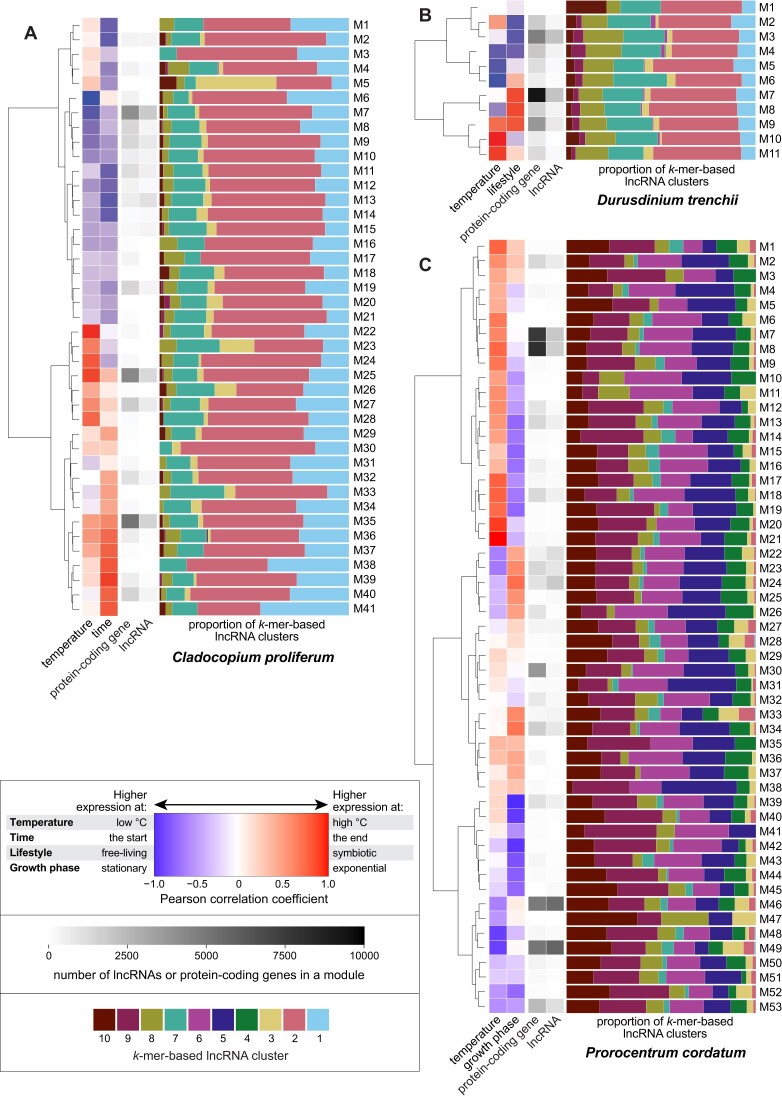
WGCNA modules of co-expressed lncRNAs, shown for (**A**) *C. proliferum*, (**B**) *D. trenchii* and (**C**) *P. cordatum*. In each panel, each row represents a WGCNA module, showing, from left to right, the Pearson correlation coefficient of eigengenes to the two external factors examined in the dataset (temperature, and time/lifestyle/growth phase), the number of genes and lncRNAs in the module, and the proportion of all lncRNAs in each *k*-mer-based cluster in stacked bar charts.

We used weighted gene co-expression network analysis (WGCNA) to assess co-expression of genes and lncRNAs in the three transcriptome datasets. A WGCNA module represents a group of similarly co-expressed genes and lncRNAs, implicating biological processes or metabolic functions that share a similar molecular response. Independently, we identified WGCNA modules for *C. proliferum* (41; Figure 5A), *D. trenchii* (11; Figure 5B) and *P. cordatum* (53; Figure 5C). The number of lncRNAs within each module shows a strong correlation with the number of genes (Spearman correlation coefficient = 0.88, 0.97 and 0.81 for *C. proliferum*, *D. trenchii* and *P. cordatum*). This observation suggests that WGCNA modules containing a greater number of genes, which likely participate in more complex biological processes and/or metabolic pathways, tend to contain a larger number of lncRNAs.

The *k*-mer profiles of lncRNAs have been associated with the repression or activation of nearby genes in eukaryotes, and with lncRNAs sharing similar protein-binding properties, linking conserved motifs to lncRNA function (63). In each dinoflagellate genome, we observed a significant correlation (Fisher's exact test, 10 000-replicate Monte Carlo simulations, *p* < 10^−5^) between the overall distribution of *k*-mer-based lncRNA clusters and the expression pattern based on WGCNA modules. Among the WGCNA modules for each taxon, we identified significant differential representation of one or more clusters in *C. proliferum* (12 of 41; Supplementary Table S2), *D. trenchii* (2 of 11; Supplementary Table S3), and *P. cordatum* (17 of 53; Supplementary Table S4). For example, in *P. cordatum*, clusters 5 and 6 were overrepresented in the two largest modules up-regulated under heat stress (M7 and M8) but underrepresented in the two largest modules down-regulated under heat stress (M46 and M49); the opposite trend was observed for clusters 2, 3 and 10 (Figure 5C and Supplementary Table S4). The conserved motifs of these implicated lncRNAs are rich in pyrimidine nucleotides (cytosine and thymidine; Supplementary Figure S3), indicating their capacity of binding to purine-rich DNA sequences via Hoogsteen (or reverse Hoogsteen) hydrogen bonds (87). This mode of action is expected for lncRNA regulation of gene expression in eukaryotes, whereby lncRNAs form a triple-helical structure by binding to the purine-rich strand of target genome regions, e.g. promoters (88).

These results clearly indicate a correlation between conserved lncRNA motifs and gene expression, and by extension, gene function in dinoflagellates. Although the specific binding sites and regulatory functions of the lncRNAs remain to be experimentally validated, considering the binding affinity of putative TFs to RNA molecules in dinoflagellates (32), our results provide insights into the potential role of ncRNAs in regulating gene expression in these microalgae.

### Gene targets of lncRNAs based on the predicted formation of triple-helical structures

We identified putative gene targets for all lncRNAs by inferring triplex interactions between each lncRNA and the upstream (promoter) region of a gene, following Buske *et al.* (69). We identified lncRNAs in *C. proliferum* (168; 1.3% of 13 435), *D. trenchii* (142; 2.0% of 7036), and *P. cordatum* (3411; 1.2% of 27 568) that putatively form a triple-helix with promoter regions of 439, 622 and 18 399 genes, respectively (Table 3). Based on the specificity of interactions between lncRNAs to genes, we categorised these lncRNAs broadly into four groups: one-to-one, one-to-many, many-to-one, and many-to-many. Among these groups, none of the lncRNAs showed many-to-one interactions with genes in all cases. Many-to-many interactions were the most abundant, comprising 58.9%, 60.6% and 82.7% of triplex-forming lncRNAs in *C. proliferum*, *D. trenchii* and *P. cordatum*, followed by the group of one-to-one interactions, targeting genes encoding distinct biological functions (Supplementary Tables S5 through S10). For instance, relative to all genes of *C. proliferum*, those implicated in many-to-many interactions (Supplementary Table S5) appear to be enriched in functions related to photoreactive repair and vesicle-mediated transport based on annotation of Gene Ontology (GO) terms, whereas those in one-to-one interactions (Supplementary Table S6) are enriched in functions related to methylation and post-replication repair.

**Table 3.** Predicted triplex interactions of lncRNAs and protein-coding genes in the three dinoflagellate datasets. DE genes in response to heat stress were shown for *C. proliferum* (32 versus 26°C), *D. trenchii* (34 versus 28°C) and *P. cordatum* (30 versus 20°C)

	*C. proliferum*	*D. trenchii*	*P. cordatum*
Number of triplex-forming lncRNAs	168	142	3411
Number of interactions	1085	2414	288 977
One-to-one	59	39	558
One-to-many	10	17	33
Many-to-one	0	0	0
Many-to-many	99	86	2820
Total number of genes	45 322	55 799	85 849
Genes with interacting lncRNAs	439 (0.9%)	622 (1.1%)	18 399 (21.4%)
Total number of DE genes under heat stress	146	11	3669
DE genes under heat stress with interacting lncRNAs	3 (2.1%)	0	904 (24.6%)

We found a greater proportion of genes interacting with lncRNAs in the free-living *P. cordatum* (21.4%), compared to the two coral symbionts *C. proliferum* (0.9%) and *D. trenchii* (1.1%) (Table 3). Moreover, this proportion is even greater for DE genes under heat stress (24.6%), and the same trend is also observed in *C. proliferum* (2.1% DE genes versus 0.9% all genes; Table 3). We did not find lncRNA interactions with DE genes that are associated with heat-stress response in *D. trenchii* (Table 3); this may be due in part to the fact that *D. trenchii* is a naturally thermotolerant species for which the distinct lifestyle (free-living versus symbiotic) stages elicits a much stronger differential molecular response than does heat stress (12,27). Although we cannot dismiss potential false positives in our prediction of triplex interactions (89), particularly among the one-to-one interactions, our results support the hypothesis of lncRNA interactions with target genes as a heat-stress response in dinoflagellates.

To assess the impact of lncRNAs on the overall transcriptional response to heat stress, we focused on *P. cordatum* for which the largest number of lncRNAs were identified. Specifically, we investigated the inferred interactions between lncRNAs and DE genes, i.e. lncRNAs for which expression is positively or negatively correlated to the DE genes based on the Spearman correlation coefficient ≥ 0.8 and *p* ≤ 0.05 following Fan *et al.* (90). We identified 2941 interactions of 435 lncRNAs implicating 527 genes; remarkably, of these interactions, 2398 (81.5%) showed a negative correlation with the expression of DE genes, suggesting a down-regulation function of interacting lncRNAs on expression of target gene. This trend aligns with the current knowledge of triplex-forming ncRNAs, most of which repress gene expression (87,91–94).

**Figure 6.**
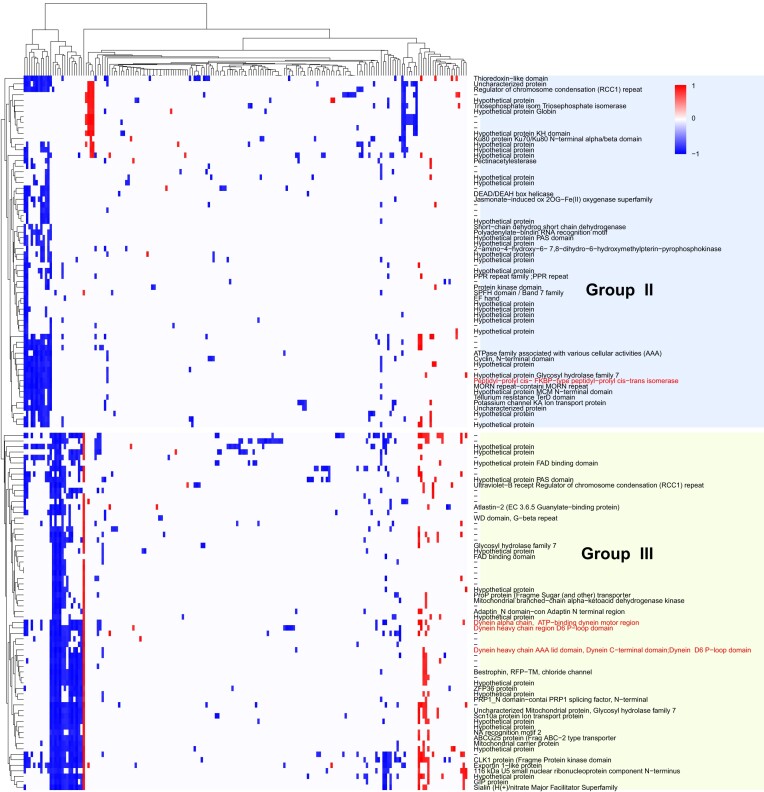
Correlation of expression of up-regulated *P. cordatum* genes under heat stress (30 versus 20°C) with the expression of their interacting lncRNAs. Each row represents a gene, and each column represents a lncRNA. Spearman correlation of expression (absolute value ≥ 0.8) is shown for each gene-lncRNA pair, with –1.0 indicating negative correlation, and 1.0 indicating positive correlation.

Among the 435 lncRNAs, 186 (42.8%) have only one gene target, implicating 137 genes, whereas the remaining 249 have multiple gene targets, forming 2755 (93.7% of 2941) interactions with 488 genes. The correlation of expression between these 488 genes and their interacting lncRNAs revealed three distinct clusters (Supplementary Figure S4): Group I (359 genes), Group II (64), and Group III (65). Group I had the smallest number of lncRNA interactions (mean 2.37), compared to Group II (11.08) and Group III (18.37). To reduce the bias of potential false positives in our interpretation of these results, we focused on Groups II and III for which a greater number of lncRNA interactions were observed (Figure 6). Interestingly, these groups consist only of genes up-regulated under heat stress. Figure 6 shows a clear pattern of negative correlation between the expression of interacting lncRNAs and the expression of up-regulated genes, and the association of lncRNA interactions to distinct gene functions of Groups II and III. Based on annotation of GO terms, the up-regulated genes in Group III largely encode functions related to microtubule-based movement and polysaccharide metabolic process, compared to those largely encoding functions related to protein metabolic processes and macromolecule modification in Groups I and II. For instance, three genes in Group III encode for the dynein heavy chain proteins (Figure 6) that are main structural and functional components of dynein motor complexes essential for intracellular transport and cell movements in eukaryotes, often encoded in multiple genes (95). Up-regulation of these genes has been associated with phosphorus deficiency in *Prorocentrum shikokuense*, indicating enhanced intracellular trafficking or cell motility due to stress (37). Our result hints at the role of lncRNAs in regulating the expression of these genes in *P. cordatum* as a heat-stress response.

### Hypothesised regulation of transcription initiation by lncRNAs in a heat-stress response

**Figure 7.**
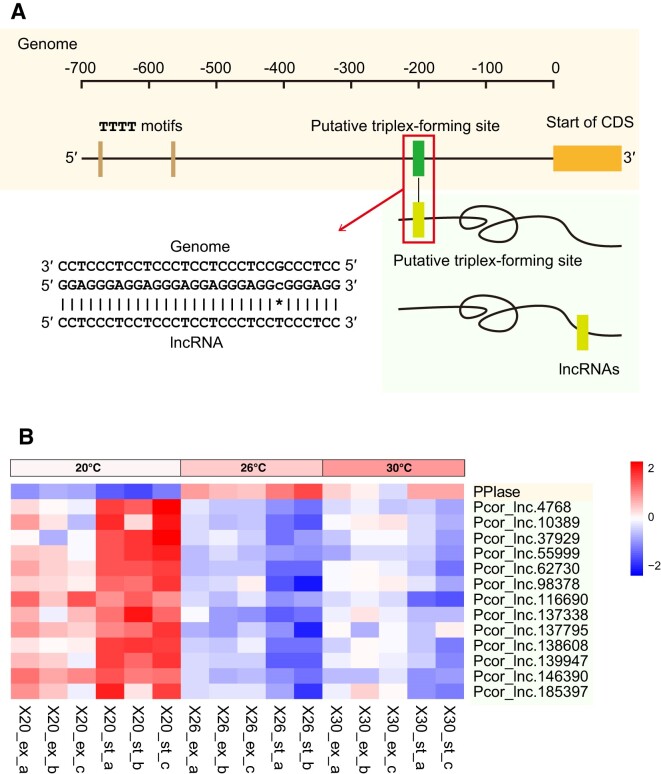
Hypothesised lncRNA regulation of transcription of the *P. cordatum* gene encoding PPIase, showing (**A**) the putative promoter region upstream of the protein-coding sequence and the predicted binding site for the formation of the triple-helical structure, and (**B**) contrasting expression pattern of the up-regulated gene encoding PPIase and the 13 interacting lncRNAs that are down-regulated.

An up-regulated gene in Group II encodes peptidyl-prolyl *cis*-*trans* isomerase (PPIase). PPIase catalyses the interconversion of the *cis* and *trans* isomers of peptidyl-prolyl peptide bonds, which is essential for proper folding of proteins that affects their stability and function (96). This up-regulated gene in *P. cordatum* is the putative target of 13 lncRNAs—that are down-regulated under heat stress (Figure 6). The predicted purine-rich binding site for these lncRNAs is located between 193 bp and 226 bp upstream of the protein-coding sequence (Figure 7A), a region hypothesised to form the triple helical structure.

Notably, a TTTT motif was identified at 680 bp and 568 bp upstream of the coding sequence, which has been described as potential core promoter motif in dinoflagellates in lieu of the typical TATA-box in eukaryotes (21), and a known binding site for the general transcription factor TFIID involved in the formation of the transcription preinitiation complex (TPC) in dinoflagellates (97). TPC is a protein complex essential for transcription of protein-coding genes in eukaryotes (98); in human, triple-helical DNA structure at a promoter region is known to yield non-functional TPC, thus inhibiting transcription (99). We hypothesise that the expression of lncRNAs interacting with the putative promoter region of the PPIase-encoding gene in *P. cordatum* would similarly impede TPC formation, thus attenuating the transcription of the PPIase gene. This gene would be transcribed when expression of the interacting lncRNAs was reduced. Importantly, PPIase is known to form a heterocomplex with the 90-kDa heat-shock protein, thereby regulating various biological processes including stress responses (100,101). This likely explains the up-regulation of this gene in *P. cordatum* during heat stress, supported by expression data across all sample replicates at 26°C and at 30°C relative to 20°C (Figure 7B), when the interacting lncRNAs were down-regulated.

### Concluding remarks

We present the first in-depth analysis of lncRNAs and their expression patterns in three dinoflagellate species. Our results, based on high-quality transcriptome and genome data, reveal that dinoflagellate lncRNAs are differentially expressed in response to stress, similar to protein-coding genes in these taxa. Conserved motifs in the lncRNAs, combined with their co-expression with protein-coding genes, suggests a link between lncRNAs and gene expression and their functional role as regulatory elements in dinoflagellates. These results demonstrate the utility of *k*-mers in analysing complex, highly divergent genomic elements. We also discovered potential gene targets whose expression may be regulated by triplex-forming lncRNAs; these lncRNAs and their proposed functions are strong candidates for experimental validation. Knowledge of RNA-protein binding and RNA-DNA binding in dinoflagellates will further elucidate the possible functional domains (i.e. conserved structural features) in lncRNAs. However, because the datasets we used were generated from RNA-Seq experiments restricted to poly-adenylated RNAs, non-polyadenylated lncRNAs and decayed lncRNAs (e.g. due to other RNA decay machineries as observed in yeasts (102–104)) were not addressed here. We assessed the potential formation of triple-helical structures as a proxy for interactions between lncRNAs and their gene targets. The functions of the lncRNAs, including their regulation of gene expression via binding to or interacting with other regulatory elements (e.g. microRNAs) (34,40), remain to be validated through more-targeted experiments. Nevertheless, our results demonstrate how the inclusion of lncRNAs in transcriptome analyses provides novel insights into the molecular mechanisms that underpin RNA-based regulation of gene expression in dinoflagellates. Analysis of these atypical genomes provide insights that may extend more broadly to other microbial eukaryotes.

## Supplementary Material

lqae016_Supplemental_Files

## Data Availability

All published transcriptome datasets were downloaded from NCBI Sequence Read Archive through BioProject accessions PRJNA723630, PRJNA723630, PRJNA295075, PRJNA609212, PRJNA508937 and PRJEB54915; see Supplementary Table S1 for detail. The genome assemblies for *C. proliferum*, *D. trenchii* and *P. cordatum* were acquired from the corresponding studies detailed in Table 1. The *de novo* assembled transcripts and lncRNAs identified for the three species are available at https://doi.org/10.48610/2692e99. Our analytic workflow for identifying putative lncRNAs from the assembled transcripts is available at https://github.com/YibiChen/LncRNAPredictor and https://doi.org/10.5281/zenodo.10558662. A genome browser for visualising the mapping of RNA-Seq reads relative to the identified lncRNAs for each genome is available at https://prorocentrum.genome.edu.au/apollo/jbrowse.
