# Supplementary material for: Genome-wide transcriptome analysis reveals the diversity and function of long non-coding RNAs in dinoflagellates
Source: NAR Genom Bioinform. 2024 Feb 10;6(1):lqae016. doi: 10.1093/nargab/lqae016 (PMC10858649; doi:10.1093/nargab/lqae016)
Supplement: lqae016_Supplemental_Files [file lqae016_supplemental_files.zip › Chen_SupplementaryFigures_S1-S4_R1.pdf]

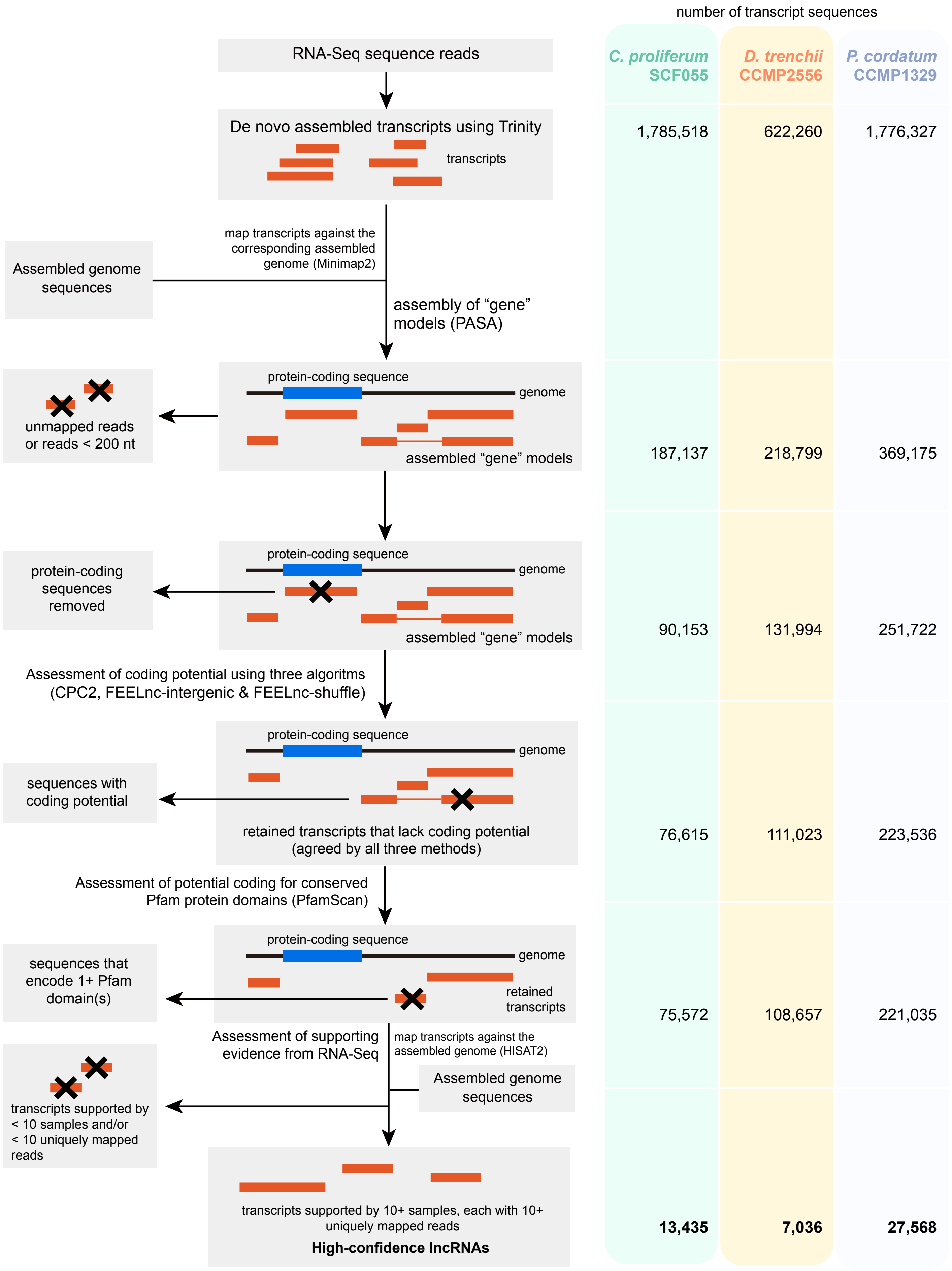

Supplementary Figure S1. Analytic workflow for identifying putative lncRNAs from RNA-Seq datasets of the three dinoflagellate taxa, indicating number of transcripts at each stage.

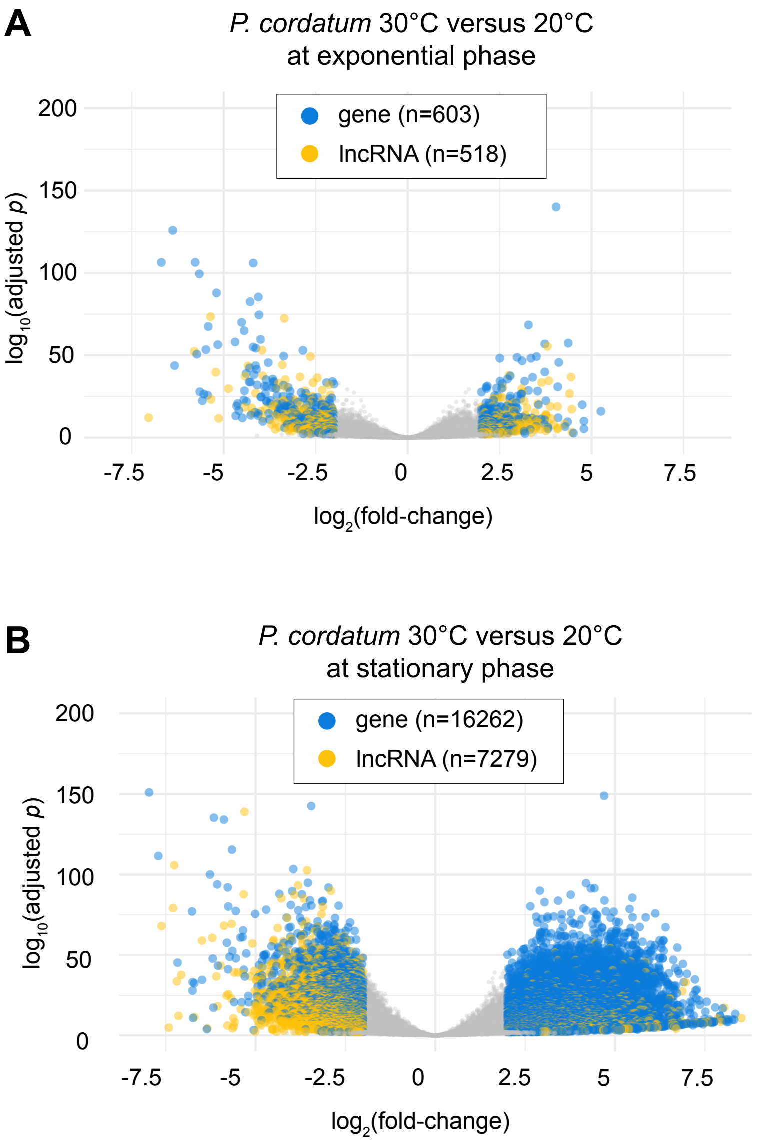

For each panel, the *x*-axis represents fold-change of transcript expression (in $\log_2$ scale), and the *y*-axis represents the significance of difference in adjusted *p*-value (in $\log_{10}$ scale). Differentially expressed genes (blue) and differentially expressed lncRNAs (yellow) were noted, and their numbers are shown for each panel.

Supplementary Figure S2. Differentially expressed genes and lncRNAs relative to heat stress for *P. cordatum* (30 versus 20°C), respectively for (**A**) exponential phase and (**B**) stationary phase.

a

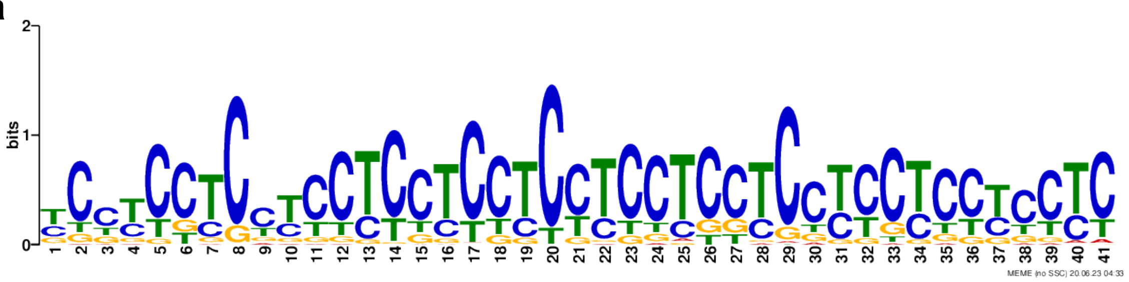

The most frequent motif in clusters 5 and 6, in M7 and M8

MOTIF YCCYCCTCCYCCTCCTCCTCCTCCTCCTCCYCCYCCYCCTC

width = 41 sites = 299 llr = 5793 E-value = 2.1e-162

b

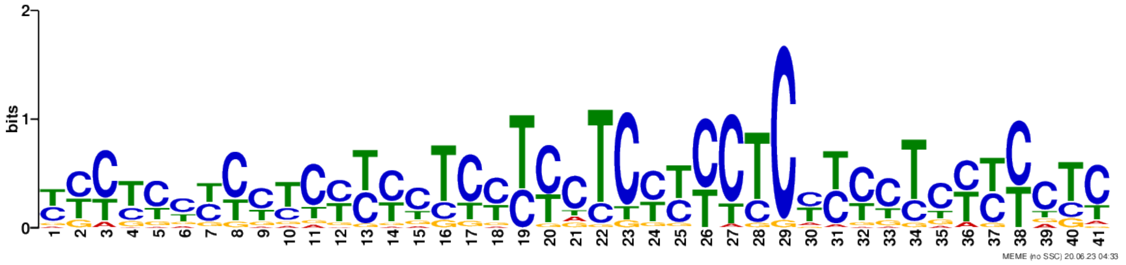

The most frequent motif in clusters 2, 3, and 10, in M46 and M49

MOTIF YYCYCYYCYYCYYYCTCYYYCTCYYYCTCYYYCTCYYYCTC

width = 41 sites = 996 llr = 15952 E-value = 2.9e-082

Supplementary Figure S3. The conserved CT-rich motifs lncRNAs in *k*-mer-based clusters that were found to be significantly overrepresented in distinct WGCNA modules.

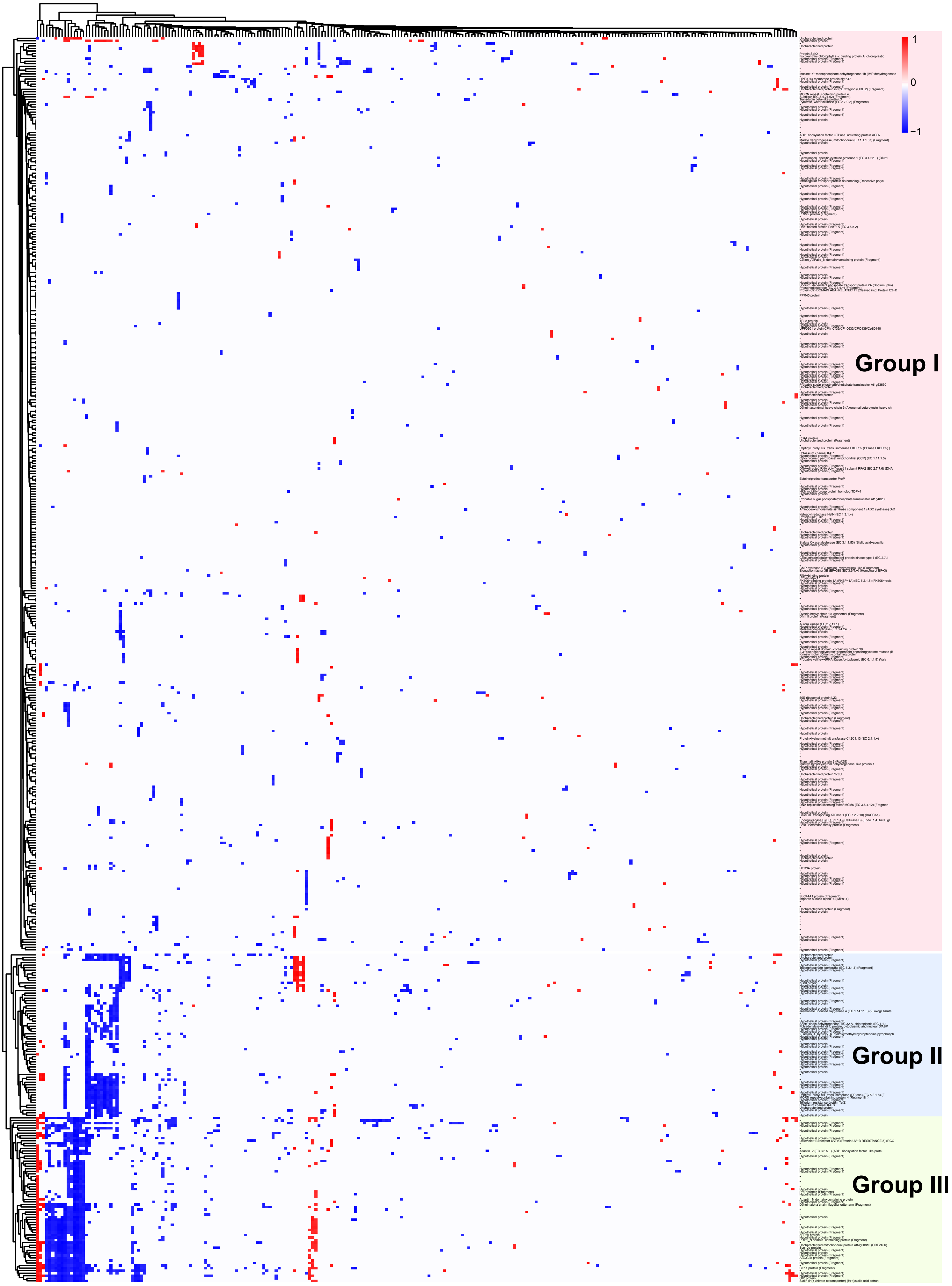

Supplementary Figure S4. Correlation of expression of differentially expressed *P. cordatum* genes under heat stress (30 versus 20°C) with the expression of their interacting lncRNAs.
